# Association Between 5-HTTLPR Polymorphism and the Risk of Autism: A Meta-Analysis Based on Case-Control Studies

**DOI:** 10.3389/fpsyt.2019.00051

**Published:** 2019-02-13

**Authors:** Hongbing Wang, Fangna Yin, Junwei Gao, Xiaotang Fan

**Affiliations:** ^1^Department of Radiotherapy Oncology, Cangzhou Central Hospital, Cangzhou, China; ^2^Clinical Laboratory, Cangzhou Central Hospital, Cangzhou, China; ^3^Department of Developmental Neuropsychology, School of Psychology, Third Military Medical University, Chongqing, China

**Keywords:** gene, polymorphism, autism, 5-HTTLPR, case-control

## Abstract

**Background:** Recently, many case-control studies have reported the association between 5-HTTLPR polymorphism and autism risk. However, the results are inconclusive and conflicting. To investigate the genetic association of 5-HTTLPR polymorphism and autism risk, we conducted a comprehensive meta-analysis based on previous case-control studies.

**Methods:** Literature search was performed through PubMed, Embase, Web of Knowledge and CNKI databases until June 27, 2018. The strength of the association was assessed by relative risk (RR) and its corresponding 95% confidence interval (CI). Fixed or random effect model was selected based on the results of heterogeneity test. Further, subgroup analyses were conducted to explore the association of 5-HTTLPR polymorphism and autism risk in different population.

**Results:** Eleven studies with 930 cases and 1234 controls were identified. Although there was a significant association between 5-HTTLPR polymorphism and autism risk under the dominant model after removing the studies causing heterogeneity, the significance did not exist after Bonferroni's correction. Subgroup analyses also showed similar results after Bonferroni's correction. In addition, there was no obvious publication bias in our meta-analysis.

**Conclusions:** Our present meta-analysis does not support a direct effect of 5-HTTLPR polymorphism on autism risk according to present results. Further analyses of the effect of genetic networks and more well designed studies with larger sample size are required.

## Introduction

Autism spectrum disorder (ASD) is defined as a group of neurodevelopmental disorders classically characterized by impaired social communication, repetitive behaviors and selective attention (1, 2). In the past decade, the number of people with a diagnosis of ASD has escalated, and prevalence rate has been exceeded 1 person in 150 worldwide (3). Accumulating evidence indicates that ASD is one of the most heritable neuropsychiatric disorders (4). Monoaminergic signaling system is thought to play an important role in some processes, such as negative emotions, impulsivity, working memory and making decisions (5–7). Recently, although some GWAS studies produced little support for monoaminergic signaling system on some psychiatric disorders, a number of studies have suggested the association between genetic polymorphisms of monoaminergic signaling system and psychiatric disorders, such as major depressive disorder, bipolar disease, schizophrenia and autism (8–11). Therefore, to explore the relation between monoaminergic signaling system and autism and further find some susceptibility genes might be a promising approach to take early intervention to autism patients.

Serotonin transporter (5-HTT) is one of the most common members in monoaminergic signaling system and belongs to the Na^+^/Cl^−^ dependent solute-carrier family of transporters (12). It could take 5-hydroxytryptamine (5-HT) up into the presynaptic neuron after 5-HT is released in synapses, which recycles 5-HT into the neurotransmitter pool. Serotonin transporter is encoded by the human solute carrier family 6 (neurotransmitter transporter), member 4 (SLC6A4) (GenBank accession number NG_011747.2), which is located on chromosome 17q11.1-17q12, containing 15 exons. The serotonin-transporter-linked promoter region (5-HTTLPR), located in the promoter region of the SLC6A4 gene, has been widely studied for its role in altering 5-HTT messenger RNA (mRNA) levels. A functional insertion-deletion polymorphism in the promoter region gives rise to two main alleles, the short (S) (14 repeats) and long (L) (16 repeats) variants (13–15). The short allele has 42 base pairs (bps) less than the long allele. In addition to the L/S alleles, although researchers also defined L_A_ and L_G_ based on a mutation of adenine to guanine in the L allele and some other variations, most clinical studies only showed results of the L/S alleles. The S/S genotype was present in the 22% of Caucasians and in 60% of Asians, whereas the L/L genotype was present in 29–43% of Caucasians, but in 1–13% of East Asians. Traditionally, the S variant is deemed to inhibit transcription and negatively affects the 5-HTT recycling from synaptic cleft (16, 17), and then, related to suicidal behavior, depression, and geriatric depression (18–20).

In 2008, Huang et al. conducted a meta-analysis using family-based studies and showed preferential transmission of the s allele of 5-HTTLPR in US mixed population samples (21). Nevertheless, they reported only two population-based studies and a formal meta-analysis of these studies could not be done. Moreover, Yang et al. found 5-HTTLPR polymorphism did not significantly affect ASD risk based on six case-control studies with obvious heterogeneity (22). However, more population-based studies explored the effect of 5-HTTLPR polymorphism on autism risk but the results were still not consistent and remained inconclusive. Based on these above, we performed the present meta-analysis according to published case-control papers to clarify the association between 5-HTTLPR polymorphism and autism risk.

## Methods

### Publication Search

To identify the relevant records which explored the association of 5-HTTLPR polymorphism with autism risk, we performed a systematic literature search of PubMed, Embase, Web of Knowledge and CNKI (Chinese National Knowledge Infrastructure) databases up to June 27, 2018. The combination of terms “SERT,” “5-HTT,” “SLC6A4” or “serotonin transporter gene”; “autism”, “autistic” or “ASD”; and “polymorphism,” “variation,” “mutation,” or “genotype” was used in order to obtain all the genetic studies on the association of 5-HTTLPR genetic polymorphism and autism. The references of retrieved studies were also examined to identify additional studies. For the related data not revealed in original articles, we tried to contact their authors.

### Inclusion and Exclusion Criteria

In our meta-analysis, records were included using the following criteria: (1) case-control studies evaluating the association between 5-HTTLPR polymorphism and autism risk; (2) the number or frequency of genotypes was provided in studies or obtained by contacting the authors; (3) records were clinical studies. The exclusion criteria were: (1) studies with insufficient information such as genotype frequency or number not obtained were excluded; (2) transmission/disequilibrium test (TDT) studies; (3) review, comment or abstract; (4) patients with genetic disorders associated with ASD were excluded. If the same population was included in previous studies, only the one with the largest sample size was included.

### Data Extraction

Data extraction was conducted independently by two investigators in order to reduce personal error. The following information from each study was recorded: first author's name, publication year, country, ethnicity, diagnostic criteria, age group, genotyping method, genotype number for cases and controls. A standardized data collection protocol was used for information collection. Discrepancies were settled by discussion and consensus.

### Statistical Analysis

We performed the meta-analysis with the allele comparison model (s vs. l), the codominant model (ss vs. ll), the recessive model (ss vs. ls/ll) and the dominant model (ss/ls vs. ll) for the unknown inherited model of autism. The pooled relative risk (RR) and its corresponding 95% confidence interval (CI) were used to evaluate the strength of association between 5-HTTLPR polymorphism and autism risk. Fixed effect model or random effect model was selected based on heterogeneity analysis, which was assessed by *I*^2^ value and Chi-square based Q-test. A significant Q statistic (*P* < 0.10) or *I*^2^ > 50% indicated heterogeneity across studies and a random effect model was used to calculate corresponding RRs; otherwise, a fixed effect model was selected. Z test was used to determine the statistical significance of RRs. In addition to overall analysis, subgroup analysis was conducted in Chinese Han population and children. To assess the stability of our results, we performed sensitivity analysis by removing each study in turn. Hardy-Weinberg equilibrium was tested based on the reported genotype frequencies among controls. To evaluate whether publication bias was a threat to the validity of our results, Begg's funnel plot (the more symmetrical the lower risk of publication bias) and Egger's test were applied. At last, Bonferroni's correction was conducted to control the type I error rate in our meta-analysis. All statistical tests were performed using STATA 12.0 software (STATA Corp, College Station, TX).

## Results

### Study Characteristics

**Figure 1:**
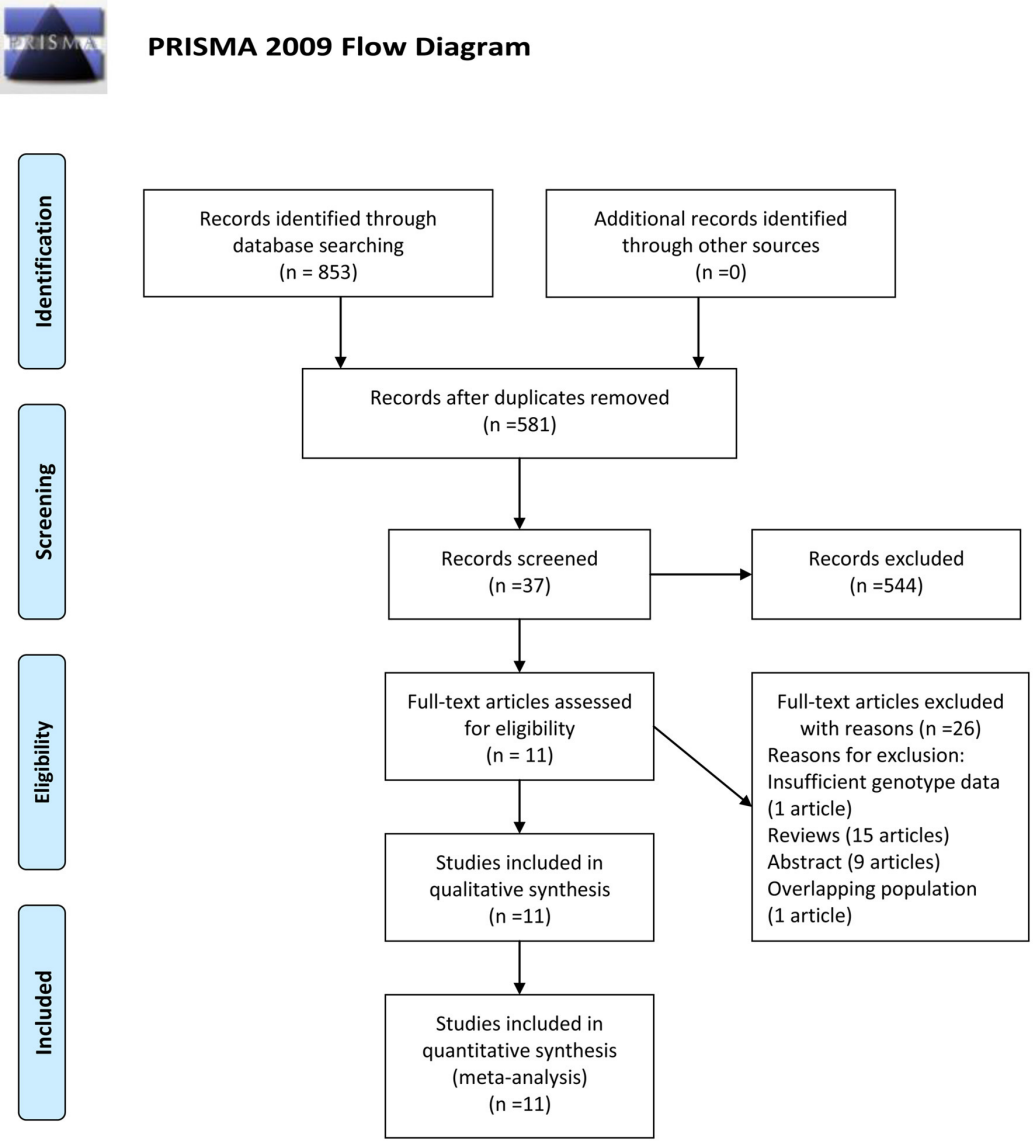
Flow diagram of study identification.

A total of 853 records were identified update to June 27, 2018 (134 from PubMed, 265 from Embase, 347 from Web of Knowledge and 107 from CNKI). Two hundred and seventy-two duplications were found in the records and 544 records were excluded for the unmatched titles or abstracts. Full text reading help removed 26 records (1 record with insufficient genotype data, 15 reviews, 9 abstracts and 1 with overlapping population) from the last 37 records. At last, 11 records (11 studies) (23–33) with 930 autism cases and 1,234 controls were included in our meta-analysis. (Flow diagram in Figure 1).

The characteristics of included studies were listed in Table 1. There were 3 studies (24, 25, 33) performed in Chinese Han population, but most study focused on mix population. There was no obvious difference in allele frequencies across ethnic groups based on present information. But we could not do subgroup analyses based on ethnicity for mix population. Most samples were children. The majority of authors selected PCR to genotyping samples. Ten studies were consistent with Hardy-Weinberg equilibrium testing. The diagnosis of autism was confirmed by the Diagnostic and Statistical Manual of Mental Disorders, 4th Edition (DSM-IV) and the Childhood Autism Rating Scale (CARS) in most studies. In addition, Faja et al. (32) did not distinguish the ss and ls genotypes in their study, so we have to record the ss+ls genotype number.

**Table 1:** Characteristics of the studies included in the meta-analysis.

**Author Publication year**	**Country**	**Ethnicity**	**Age group**	**Diagnostic criteria**	**Genotyping method**	**Sample size**		**Autism**			**Control**			**HWE**
						**Autism**	**Control**	**ss**	**ls**	**ll**	**ss**	**ls**	**ll**	
Faja et al. (32)	USA	Mix	Adult	DSM-IV-TR	PCR	27	25	13^a^		9	19^a^		4	NA
Zhang et al. (33)	China	Han	Pediatric	DSM-IV	PCR-RFLP	58	59	9	45	4	23	25	11	Yes
Meguid et al. (31)	Egypt	NA	Pediatric	DSM-IV and CARS	PCR	20	20	0	4	16	0	0	20	Yes
Jaiswal et al. (30)	India	NA	Pediatric	DSM-IV-TR	PCR	169	168	58	84	27	70	75	23	Yes
Nyffeler et al. (29)	Switzerland	Caucasians	Pediatric	ADOS	TaqMan	76	97	24	38	14	22	51	24	Yes
Tassone et al. (28)	USA	Mix	Pediatric	ADI-R and ADOS	PCR	189	167	40	97	52	40	74	53	Yes
Arieff et al. (27)	South Africa	Mix	Pediatric	JSAIS and SSAIS	PCR	109	342	32	20	57	16	116	210	Yes
Long et al. (26)	Brazil	Mix	Pediatric	DSM-IV-TR and CARS	PCR	151	179	33	68	50	40	83	56	Yes
Tian et al. (25)	China	Han	Pediatric	CARS	PCR	35	60	25	8	2	41	13	6	Yes
Li et al. (24)	China	Han	Pediatric	DSM-IV	PCR	24	25	13	3	8	10	0	15	Yes
Zhong et al. (23)	USA	Mix	NA	DSM-IV	PCR	72	92	9	31	32	20	44	28	Yes

NA, not available;

a *represents the number of ls+ss genotype; HWE, Hardy-Weinberg equilibrium*.

### Quantitative Data Synthesis

**Figure 2:**
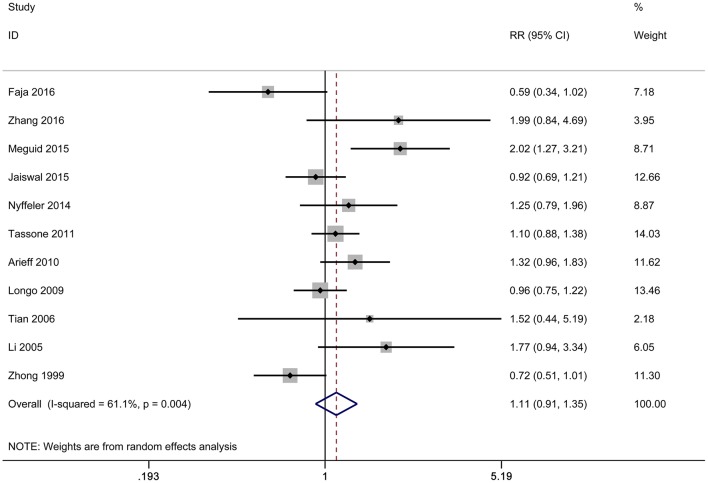
Forest plot of autism associated with 5-HTTLPR polymorphism under the dominant model.

In our present meta-analysis, eleven studies with 930 cases and 1,234 controls determined the effect of 5-HTTLPR l/s polymorphism on autism risk. Results were shown in Table 2. There were no significant associations between 5-HTTLPR polymorphism and autism risk under each genetic model for overall analysis (for s vs. l: *RR* = 1.13, 95%CI 0.95–1.34, *P* = 0.163; for ss vs. ll: RR = 1.20, 95%CI 0.82–1.78, *P* = 0.351; for ss vs. ls/ll: RR = 1.08, 95%CI 0.73–1.58, *P* = 0.704; for ss/ls vs. ll: RR = 1.11, 95%CI 0.91–1.35, *P* = 0.289;) (Table 2, Figure 2). Nevertheless, in the stratified analyses of Chinese Han population and children, significant associations were observed under the dominant model (Table 2). However, this polymorphism was no longer significantly associated with autism under the dominant model after Bonferroni's correction (for Chinese Han population: *P* = 0.272; for pediatrics: *P* = 0.512).

**Table 2:** Summary of meta-analysis results.

		**Test of association**				**Heterogeneity**		
**Groups**	**Studies**	**RR[95%CI]**	***p*-value**	**Model**	***Z***	***x*^**2**^**	***p-*value**	***I*^**2**^(%)**
**TOTAL STUDIES**								
s vs. l	10	1.13 [0.95–1.34]	0.163	RE	1.40	47.90	0.000	81.2
ss vs. ll	9	1.20 [0.82–1.78]	0.351	RE	0.93	53.4	0.000	85.0
ss vs. ls/ll	9	1.08 [0.73–1.58]	0.704	RE	0.38	84.62	0.000	90.5
ss/ls vs. ll	11	1.11 [0.91–1.35]	0.289	RE	1.06	25.7	0.004	61.1
**SUBGROUP ANALYSES**								
**CHINESE HAN**								
s vs. l	3	1.13 [0.80–1.60]	0.502	RE	0.67	4.86	0.088	58.8
ss vs. ll	3	1.42 [0.85–2.37]	0.179	FE	1.34	0.50	0.777	0
ss vs. ls/ll	3	0.90 [0.48–1.66]	0.725	RE	0.35	6.72	0.035	70.3
ss/ls vs. ll	3	1.80 [1.11–2.92]	0.017	FE	2.38	0.13	0.939	0
**PEDIATRICS**								
s vs. l	9	1.18 [0.99–1.41]	0.065	RE	1.85	41.4	0.000	80.7
ss vs. ll	8	1.32 [0.88–1.97]	0.181	RE	1.34	46.4	0.000	84.9
ss vs. ls/ll	8	1.14 [0.76–1.70]	0.537	RE	0.62	80.7	0.000	91.3
ss/ls vs. ll	9	1.22 [1.02–1.45]	0.032	RE	2.15	14.8	0.063	45.9
**REMOVING STUDIES OUTSIDE THE BOUNDARIES IN GALBRAITH PLOTS**								
s vs. l	7	1.00 [0.93–1.08]	0.943	FE	0.07	8.83	0.183	32.1
ss vs. ll	6	1.10 [0.92–1.33]	0.298	FE	1.04	3.61	0.607	0
ss vs. ls/ll	6	1.00 [0.86–1.15]	0.957	FE	0.05	5.31	0.379	5.9
ss/ls vs. ll	8	1.13 [1.00–1.28]	0.048	FE	1.98	8.83	0.265	20.7

*RE, random effects model; FE, fixed effects model; HWE, Hardy-Weinberg equilibrium*.

### Heterogeneity Analysis

**Figure 3:**
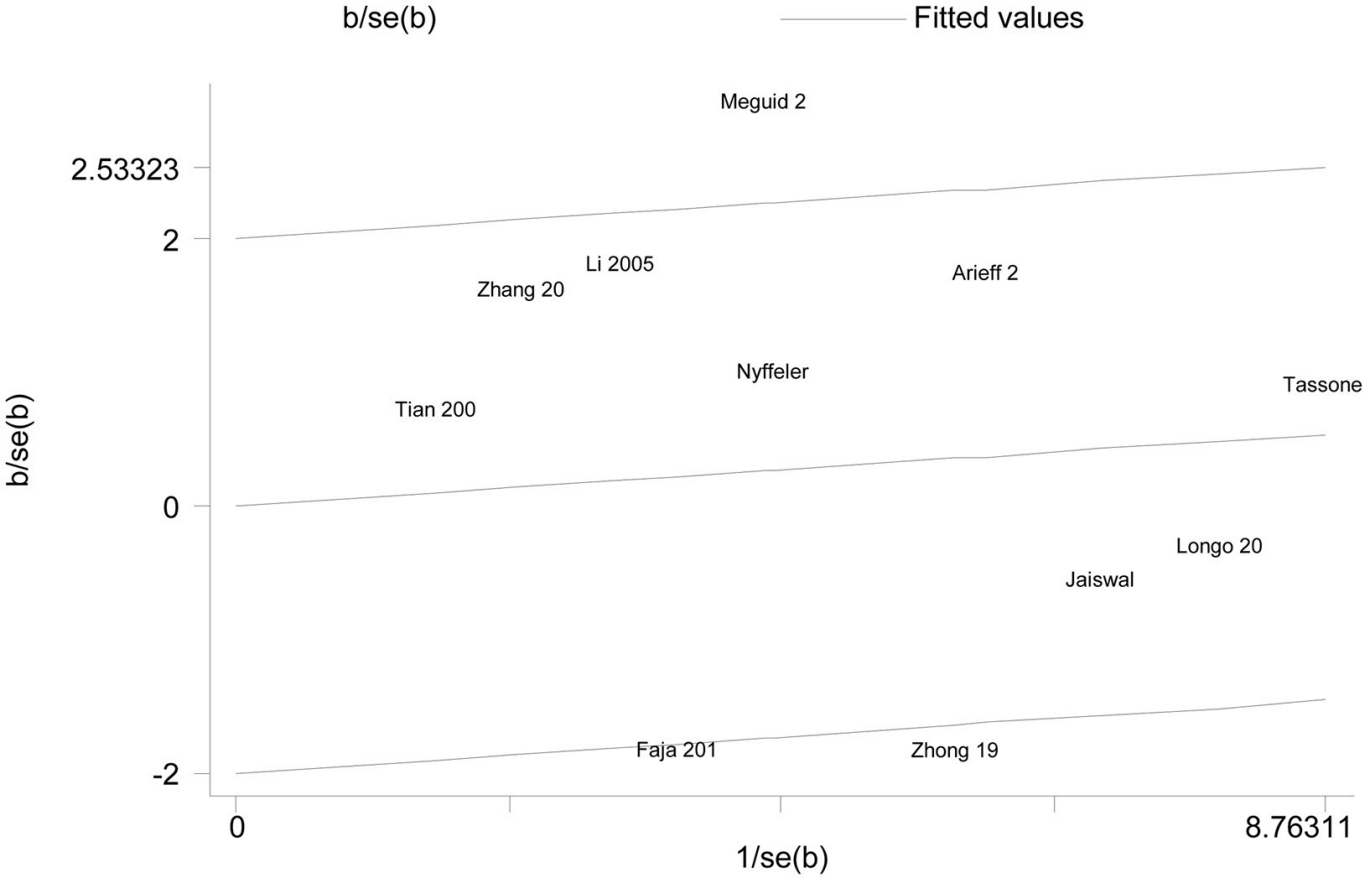
Galbraith plot of autism associated with 5-HTTLPR polymorphism under the dominant model.

**Figure 4:**
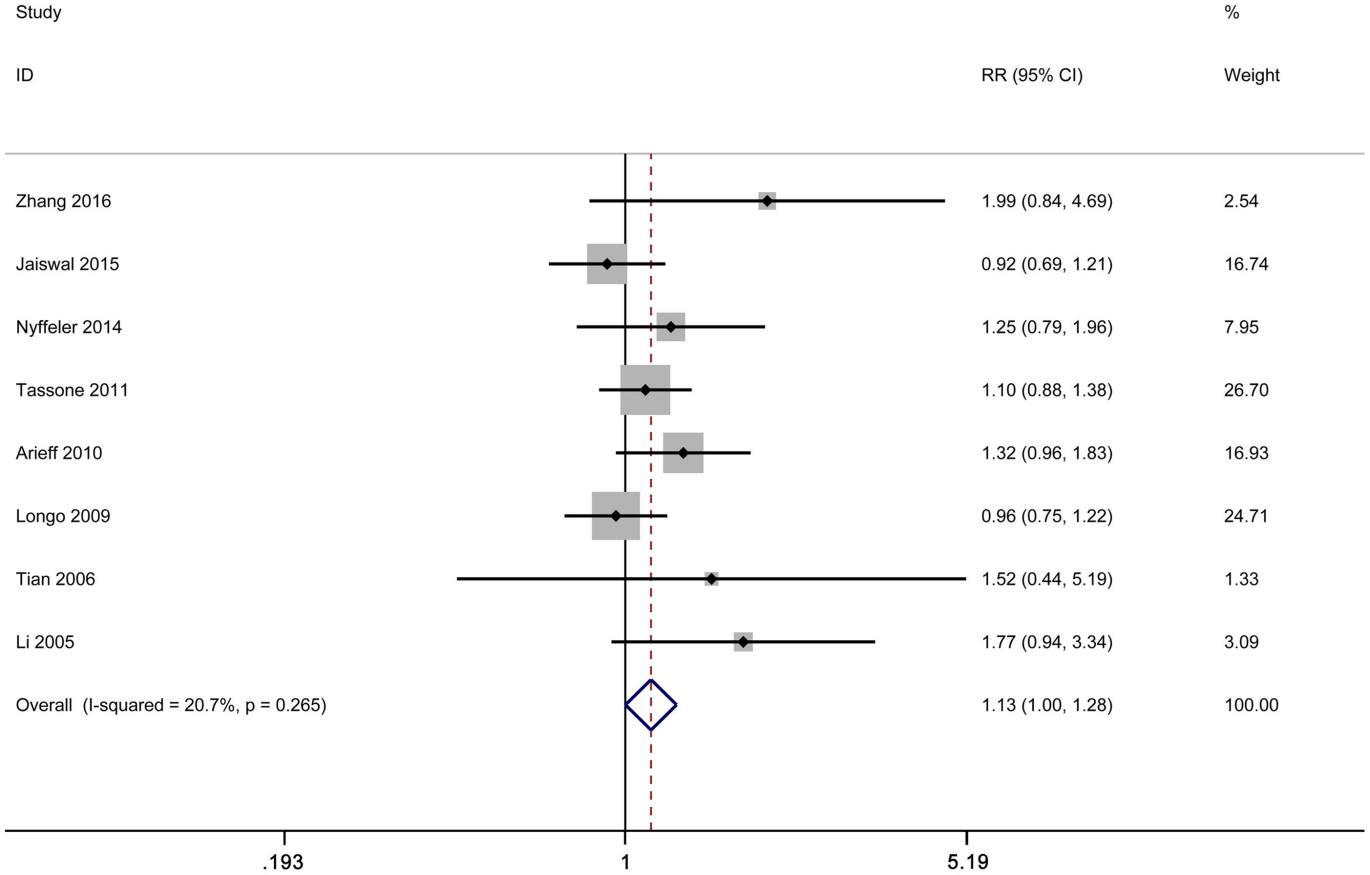
Forest plot of autism associated with 5-HTTLPR polymorphism under the dominant model after removing the studies conducted by Faja et al. (32), Meguid et al. (31), and Zhong et al. (23).

We employed *I*^2^ value and Chi-square based Q-test to evaluate heterogeneity across selected studies. Obvious heterogeneity was observed in overall comparisons under all genetic models (Table 2). Thus, random effect model was used to synthesize the data for our analysis. Even though subgroup analyses were done, significant heterogeneity across studies was still existed in our results. To further evaluate the source of heterogeneity, Galbraith plot was applied in our meta-analysis. We found that there were three studies respectively affecting the source of heterogeneity under each genetic model [s vs. l:(23, 27, 31); ss vs. ll: (23, 27, 30); ss vs. ls/ll:(27, 30, 33); ss/ls vs. ll: (23, 31, 32)] (Figure 3). After removing these studies, results indicated that no significant heterogeneity was found in any genetic model. Moreover, we found that 5-HTTLPR l/s polymorphism could increase autism risk under the dominant model (Figure 4). Nevertheless, this association did not exist in our meta-analysis after Bonferroni's correction (*P* = 0.768).

### Sensitivity Analysis

To examine the influence of each study on the overall RRs, we carried out sensitivity analysis by excluding one individual study from the overall pooled analysis sequentially. Our results of sensitivity analysis revealed that the pooled RRs and its corresponding 95% CIs were generally similar when we removed any study under all the four genetic models. Hence, our results were relatively stable and credible.

### Publication Bias

**Figure 5:**
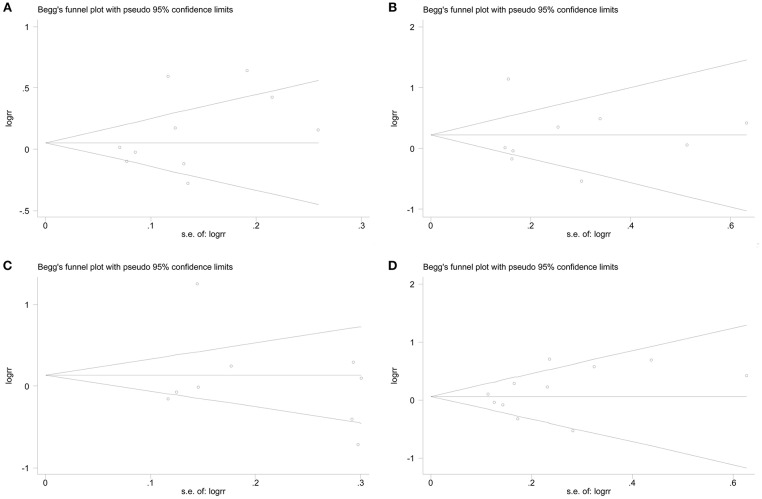
Funnel plots of 5-HTTLPR polymorphism and autism to assess publication bias under different models. **(A)** The allele comparison model; **(B)** the codominant model; **(C)** the recessive model; **(D)** the dominant model.

Begg's funnel plot and Egger's test were conducted to assess the publication bias qualitatively and quantitatively. Although the shape of the funnel plots was slight asymmetry (Figure 5), the results of Egger's test did not present any clear evidence of obvious publication bias under any genetic model (for s vs. l: *P* = 0.212; for ss vs. ll: *P* = 0.835; for ss vs. ls/ll: *P* = 0.736; for ss/ls vs. ll: *P* = 0.294).

## Discussion

In the past few decades, epidemiological studies have shown that environmental factors, such as air pollution, heavy metals, pesticides, psychological stress and nutritional factors, have an impact on the development of ASD (34). In addition, more and more evidence have suggested genetic factors may also play important roles in the development of ASD (35). Although these factors may result in abnormal development of ASD brain, the mechanism was still not completely understood. Recent studies have revealed that the imbalance of excitatory/inhibitory (E/I) system may be a crucially pathophysiological change during the development of ASD (36). All factors controlling the formation and function of excitatory and inhibitory synapses may affect the E/I system. It is well known that 5-HTT (an integral membrane protein of 630 amino acids, containing 12 transmembrane domains) could regulate the concentration of 5-HT in synapses and further change the excitatory of synapses. Therefore, the expression of 5-HTT may make an effect on the development of ASD. As the gene encoding 5-HTT, the SLC6A4 plays an important role in regulating serotonin across the serotoninergic system. Polymorphisms in the 5-HTTLPR of this gene were reported to be associated with varying degrees of transcriptional activity, and further lead to varying degrees of protein formation (17). It has been shown that 5-HTTLPR L/S alleles may result in an alteration in 5-HT and 5-HTT early in brain development, possibly affecting the development of ASD. Although many clinical studies were tried to explore the association between 5-HTTLPR polymorphism and ASD risk, the result were inconsistent.

Our meta-analysis represents a comprehensive and systematic assessment of the relation of 5-HTTLPR polymorphism with autism risk. So far, there are about eleven population based studies reported to evaluate the association between 5-HTTLPR polymorphism and autism risk. However, the results were inconsistent. A previous meta-analysis conducted by Huang et al. only explored the effect of this polymorphism based on families in 2008 (21). Compared with the previous one, our meta-analysis presented the results based on case-control studies. In addition, we examined the pooled RRs and its corresponding 95% CIs under four genetic models for the unknown inherited model. Thus, we believe that our meta-analysis explore the effect of 5-HTTLPR polymorphism on autism risk in a different way.

In our present meta-analysis, although the pooled RRs and 95%CIs under different comparison models did not show strongly significant association of 5-HTTLPR polymorphism with autism risk in overall populations under four genetic models, heterogeneity across studies may have a significant impact on results. Galbraith plots showed some studies may cause heterogeneity. After removing those studies, we found an obvious association between 5-HTTLPR polymorphism and autism risk under the dominant model. However, the association was not found under the dominant model after Bonferroni's correction. In addition, subgroup analyses on children and Chinese Han also exhibited the similar results under the dominant model. Compared with previous results, it may be caused by the limitation of population. Therefore, we hypothesized that 5-HTTLPR polymorphism may not have a direct effect on the risk of autism, which was in consistent with some recent GWAS studies. Taken together, more studies are needed to evaluate the effect of this polymorphism on autism.

Some limitations in our meta-analysis should be pointed out. Firstly, the number of studies and samples in our meta-analysis was limited for the insufficient information of some studies and unpublished records, which may influence the synthesized RRs. Secondly, autism was a complex disease and many factors may contribute to the development of it. We could only discuss the factor of ethnicity for the limited information of studies. Thirdly, some TDT studies were not included in our study to eliminate family factors. Fourthly, although we find no obvious difference in allele frequencies across ethnic groups based on present information, studies with more information about population, especially Chinese Han, should be conducted to further demonstrate our conclusion. Finally, the lack of information about the tri-allelic model of gene made us fail to analyze our data by using a tri-allelic model.

In conclusion, the present study suggested that the 5-HTTLPR polymorphism may not be associated with the risk of autism. Further analysis of studies based on larger sample size and homogeneous autism patients is required to explore the association between the 5-HTTLPR polymorphism and autism risk.

## Author Contributions

JG and XF conceived the study. HW and FY collected the data and drafted the manuscript.

### Conflict of Interest Statement

The authors declare that the research was conducted in the absence of any commercial or financial relationships that could be construed as a potential conflict of interest.
